# Evaluation of the therapeutic effect of very small stem cells from peripheral blood on the treatment of Premature Ovarian Failure: A pilot study

**DOI:** 10.5935/1518-0557.20240021

**Published:** 2024

**Authors:** Nasrin Saharkhiz, Nazanin Hajizadeh, Jahan Saheb Alkhafaji, Mohammad Hossein Mohammadi

**Affiliations:** 1 Department of Obstetrics, Shahid Beheshti University, Tehran, Iran

**Keywords:** premature ovarian failure (POF), very small stem cell, ovarian reserve, pilot study

## Abstract

**Objective:**

Premature ovary failure (POF) is a severe health condition with multiple negative outcomes, which deteriorate a patient’s life. The current study aimed to evaluate the therapeutic effect of mesenchymal stem cells (MSCs) derived from peripheral blood in the treatment of women with the POF background.

**Methods:**

The current study was a pilot study carried-out on women younger than 40 with premature ovarian failure. Study participants underwent 4-months cell therapy using Mesenchymal stem cells extracted from peripheral bloods. Serum level of Follicle-stimulating hormone (FSH), Estradiol (E2), Anti-mullerian hormone (AMH), and Antral follicle count (AFC) were the main investigated outcomes that were assessed at baseline, month two and month four of the very small stem cell intervention.

**Results:**

Average serum level of FSH was 45.0 (12.1) mIU/mL at baseline and continually decreased during the study and reached 33.2 (12.4) mIU/mL in the fourth month. The average AMH level was 0.10 ng/mL prior to the intervention and increased to 0.13 ng/mL in the 2nd month and 0.15 ng/mL in the fourth month. The level E2 was 85.7 (23.6) pg/ml on average at baseline, while the average E2 reduced to 77.2 (25.6) pg/ml in the fourth month. Average number of AFC was 2.0 (0.8) at baseline. We observed a gradual increase in the second month (Mean AFC=2.2) and after four months it increased to 3.1 (1.8) as the highest menstrual restoration and pregnancy was observed in 10% of our study participants.

**Conclusions:**

MSCs could significantly improve hormone secretion in women with POF. Implantation of MSCs in women with POF background was associated with an increase in AMH and AFC, while it downed the serum level of E2 and FSH. MSCs could also lead to menstrual restoration and pregnancy in women with POF.

## INTRODUCTION

Premature ovarian failure (POF) is a common endocrine disease causing female infertility that it is characterized by high gonadotropin expression [follicle-stimulating hormone (FSH) ≥ 40 mIU/mL], low estradiol (E2) expression, and follicular dysplasia in women aged less than 40 years (Rudnicka *et al*., 2018). POF is associated with impaired ovarian function leading to either hormonal disorders (hypoestrogenism) or loss of residual follicles that consequently increase the possibility of menstrual abnormalities, pregnancy failures, and impaired health-related quality of life. The POF is almost a rare outcome in a general population (prevalence 1%) (Coulam *et al*., 1986). However, older ages (35-40 year), family history, cigarette smoking, cancer treatment (chemotherapy and radiation) (Kenney *et al*., 2001) and genetic agents are the POF risk factors (Rudnicka *et al*., 2018; Franić-Ivanišević *et al*., 2016).

POF is a severe health condition with multiple negative outcomes deteriorating patients’ life (Rudnicka *et al*., 2018). Therefore, several therapeutic approaches like stem cell therapy are introduced to treat different modalities of POF (Li *et al*., 2010; Liu *et al.*, 2014a). Mesenchymal stem cells (MSC) could be extracted from different sources including adipose, human endometrium, human amniotic, fluid cells, human placenta, human bone marrow, and menstrual blood (Fu *et al*., 2021). Previous studies reported promising outcomes for various types of MSCs in terms of improvement of ovarian function in rats with POF. According to available evidence, MSCs play a critical role in the proliferation and repairing damaged tissues (Manshadi *et al*., 2019; Phinney & Prockop, 2007; Liu *et al*., 2014a). Local stimuli like inflammatory cytokines, ligands of Toll-like receptors, and hypoxia have strong interaction with MSCs that lead to generating a large amount of growth factors that are crucial in tissue regeneration (Castro-Manrreza & Montesinos, 2015; Dabrowska *et al*., 2019; Wei *et al*., 2013). They can also modulate the expression level of ovarian hormones to the normal level and also increase the number of follicles (Manshadi *et al*., 2019). However, most of the available evidence is limited to animal studies and rat models and there is lack of data regarding the efficacy of MSCs in humans. Moreover, in most of the previous studies, MSCs harvested from the placenta, bone marrow, and amniotic fluids and the MSCs extracted from peripheral blood were rarely investigated. The extraction of MSCs from peripheral blood is more convenient than other types of MSC (Borlongan *et al*., 2010; Mou *et al*., 2013). Higher in-vitro survival and proliferation rate turn them into an appropriate and safe agent for cell therapy (Fu *et al*., 2021; Manshadi *et al*., 2019). The current study aimed to evaluate the therapeutic effect of MSCs derived from peripheral blood in the treatment of women with the POF background.

## MATERIALS AND METHODS

### Study participants

The current study was a pilot study carried-out on women younger than 40 years of age with premature ovarian failure who were referred to our infertility center in 2022. The current study was reviewed and approved by the Review Board and Committee, and informed consent was completed for each study participant with age<40 and premature ovarian failure (POF) (with amenorrhea that occurred in at least four consecutive previous cycles and were confirmed with a high serum FSH level). Primary amenorrhea, history of severe drug allergy or autoimmune disease, severe systemic disorders such as diseases of the cardiovascular system, liver, kidney and hematopoietic systems, family history of severe genetic disease of benign or malignant ovarian tumors or endometrioma, infectious diseases such as positive antibodies against HIV or syphilis and viral hepatitis were the exclusion criteria. We also excluded patients with abnormal liver function, abnormal prolactin and thyroid function.

### Intervention

Patients with inclusion criteria received 5mg per kg body weight of subcutaneous GCS-F per day for five consecutive days. Then, all the participants were monitored using a blood cell count test until the last day of injection before sampling. Three hours after the injection of the last dose of GCS-F, 120cc blood samples were taken from each patient and placed under apheresis. The laparoscopy-guided procedure was performed to inject 2cc into the navel of the ovary due to the small size of the ovaries and difficulty in vaginal access.

### CD34 positive PBSCs (peripheral blood stem cells) mobilization Protocol

Mobilization of stem cells is currently most commonly achieved by the administration of cytokines alone or preceded by chemotherapy. G-CSF (or filgrastim, Neupogen, Amgen) is the most commonly used cytokine in this setting. G-CSF stimulates neutrophil production and maturation, and it induces the release of various proteases into the marrow, which disrupt the adhesion of CD34 stem cells to the marrow stroma, facilitating their subsequent release into the PB. The default dose of G-CSF at our institution is 10 mg/kg/day for 5 days. PegaGen® is the brand name of Pegfilgrasti (cinnagen company, Iran). PegaGen^®^ is supplied in 6 mg/0.6 mL prefilled syringes and autoinjectors (Physioject™). This drug is available in packs containing 1 sterile pre-filled syringe or Physioject™ and a patient information leaflet. The dose given once a day, enables stem cell mobilization after the last dose of cbc is rechecked, and if there is no side effect, with no problem concerning the isolation.

### CD34 positive PBSCs (stem cells) Isolation technique from the peripheral blood

For each patient, 120 mL of peripheral blood was collected in a 4 centrifugable syringe device (Rooyagen PBSCs Kit, Arya Mabna company, Iran), each device containing 5 ml of 3.8% (wt/vol) sodium citrate. Subsequently, the blood was centrifuged for 12 minutes, in the first stage,1600 RPM (450 G). In the next step, the supernatant plasma part was drawn using another 20 ml syringe and transferred to a number eight, 10 ml VBCT sterile tube and centrifuged at 3,500 RPM (2,000 G) in the second round. After finishing the centrifuge, the upper soup was discarded and only 2 cc of plasma remained, then the buffy coat resuspended to the remaining plasma. However, it should be noted that the buffy coat pellet part contained leukocytes, platelets and CD34 positive PBSCs.

### Outcome assessment

We collected data on age, body mass index (BMI), type and duration of infertility as baseline characteristics of the study participants. Serum level of Follicle-stimulating hormone (FSH), Estradiol (E2), and Anti-mullerian hormone (AMH) were the main investigated outcomes that were assessed at baseline (prior the intervention), month two and four of the very small stem cell intervention. We also measured the number of Antral follicle count (AFC) at baseline, 2^nd^ and 4^th^ month of intervention. Menstrual restoration and pregnancy were the secondary outcomes that were measured during the follow-up examinations in the second and fourth months.

### Statistical analysis

We described the data using mean and standard deviation. We also provided number and proportion for dichotomous variables. We checked the normality distribution using the Shapiro-Wilk test. We assessed the Whitin-group variability over the study period using repeated ANOVA measurements. All statistical analyses were performed using the Stata software (Ver 17.0, StataCorp, College Station, Texas, USA) and a *p*-value<0.05 was considered significant.

## RESULTS

The current study was performed on 20 patients with premature ovarian failure background who underwent one-month treatment with very small steam cells from peripheral blood. Average age of the study participants was 36.2 (1.6) years. Average BMI was 26.6 (3.0) kg/m^2^ and 60.0% of the study participants had primary infertility. Moreover, average duration of the infertility was 4.0 (1.7) years (Table 1).

**Table 1:** Study participants baseline characteristics.

Characteristics	N=20
Age, mean (SD)	36.2 (1.6)
BMI, mean (SD)	26.6 (3.0)
Type of infertility, n (%) Primary Secondary	12 (60.0%) 8 (40.0%)
Duration of infertility, mean (SD)	4.0 (1.7)

We assessed serum level of three hormones including FSH, AMH, and E2 at baseline and in two follow-up time in 2^nd^ month and 4^th^ month of therapeutic course, as well. Average serum level of FSH was 45.0 mIU/mL (12.1) at baseline and continually decreased in the second month and in month four, and reached 44.3 (17.4) mIU/mL, and 33.2 (12.4) mIU/mL, respectively. The within-group variability was statistically significant (*p*-value<0.001). There was an upward trend in serum level of AMH where the average AMH level was 0.10 prior to the intervention and increased to 0.13 in the 2^nd^ month; and 0.15 in the fourth month. The observed trend was statistically significant (*p*-value<0.001). We also depicted a decreasing pattern in the serum level of E2. The E2 level was 85.7 (23.6) pg/ml on average at baseline; however, after two months it reached 81.2 (22.7) pg/ml, and the average E2 was 77.2 (25.6) pg/ml in the fourth month. The observed change was statistically significant (*p*-value=0.001) (Table 2).

**Table 2:** The trend of FSH, AMH, and E2 hormone serum levels over the one-month treatment period with very small stem cells from peripheral blood in patients with premature ovarian failure.

Type of hormone	Pre-treatment	2^nd^ month	4^th^ month	*p*-value
FSH mIU/mL, Mean (SD)	45.0 (12.1)	44.3 (17.4)	33.2 (12.4)	<0.001
AMH ng/mL, Mean (SD)	0.1 (0.1)	0.13 (0.1)	0.15 (0.1)	<0.001
E2 pg/mL, Mean (SD)	85.7 (23.6)	81.2 (22.7)	77.2 (25.6)	0.001

* Data were provided as mean and standard deviation.

**Figure 1:**
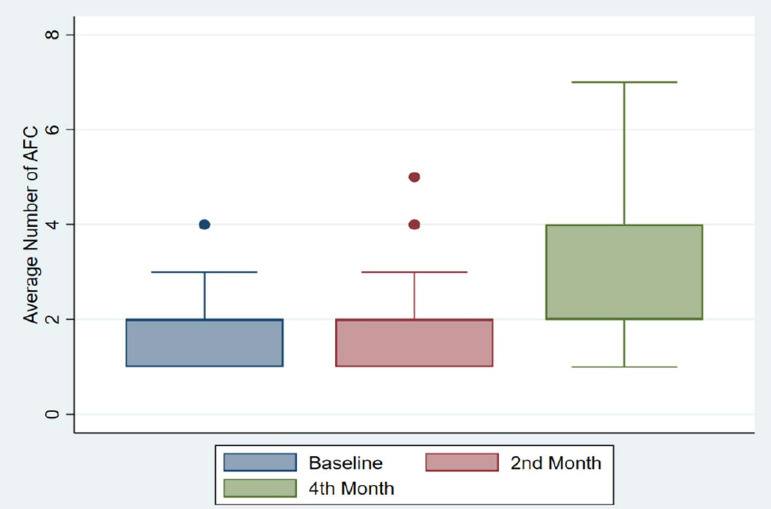
Change in the average number of AFC during the four-month treatment with very small stem cells from the peripheral blood in patients with premature ovarian failure.

Average number of AFC was 2.0 (0.8) at baseline. There was a gradual increase in the second month (Mean AFC=2.2), and after four months it increased to 3.1 (1.8) as the highest. The increase we found was statistically significant (*p*-value<0.001) (Figure 1).

**Figure 2:**
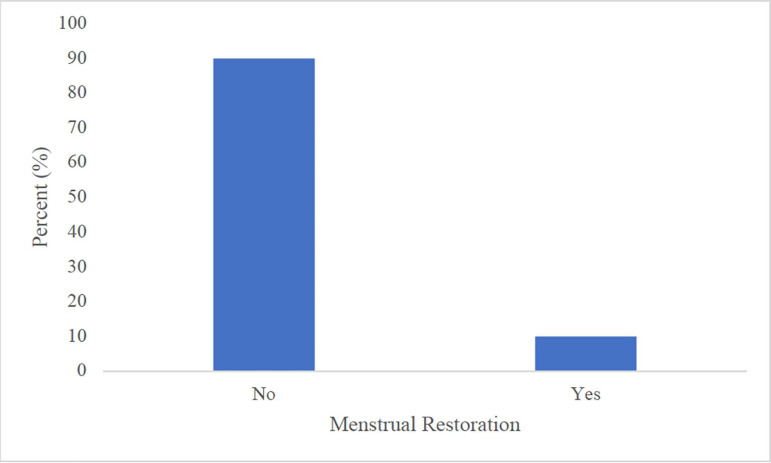
Ratio of patients with and without menstrual restoration after the four-month treatment with very small stem cells from peripheral blood in patients with premature ovarian failure.

There was also menstrual restoration in 10% of our study participants after a 4-month MSC intervention (Figure 2).

## DISCUSSION

The present study aimed to investigate the therapeutic effect of very small stem cells from the peripheral blood on women with POF. We assessed the effect of very small stem cells on serum levels of E2, FSH, and AMH; and also, the effect of stem cell treatment on the number of AFC.

Our data showed that the therapeutic approach with very small stem cells extracted from the peripheral blood could significantly increase the serum level of AMH; while its effect on FSH and E2 was inverse and led to a significant reduction in the serum level of E2 and FSH. Moreover, the stem cell intervention was associated with an increased average AFC. Proportion of menstrual restoration and pregnancy was 10% and it was observed in 2 patients. Similar findings were reported by previous studies. Several studies have shown promising outcomes for different types of mesenchymal stem cells (MSC) regardless of their source of extraction in terms of recovering ovarian function (Fu *et al*., 2021; Liu *et al*., 2014a; Manshadi *et al.*, 2019). Upregulation of AMH and increasing expression levels of FSH receptor is the proposed mechanism for this improvement, which causes granulosa cell apoptosis and follicular atresia (Yoon, 2019). A rat model study confirming our findings showed that a 30-day stem cell treatment could increase the levels of AMH, and AFC. They also showed that such treatment could reduce FSH to near normal levels (Liu *et al*., 2014b; Besikcioglu *et al*., 2019). Similarly, we observed that after four months of stem cell treatment, the FSH level came down to less than 40 mIU/mL. It is argued that stem cells might repair ovarian function in rats with POF through the overexpression of miR-21 and targeting phosphatase, and tension homolog deleted on chromosome ten (PTEN) and recombinant human programmed cell death 4 (PDCD4) (Fu *et al*., 2017; Yang *et al*., 2020).

Blood-derived stem cells were rarely used to improve ovarian function and there is limited evidence regarding their effects on POF. However, recent studies have shown that blood-derived stem cells could be used for tissue repair as they could impose a higher proliferation rate (Liu *et al*., 2014a). In the study conducted by Manshadi *et al*. (2019), the application of blood-derived stem cells in rats with POF led to improved hormone secretion as they could be localized in the GC layer of immature follicles. According to their findings, the transplantation of MSC increased the expression level of ovarian granulose cell-specific including AMH, FST, and FSHR up to the normal level leading to an increase in the number of follicles. They also showed improvements in ovarian follicular structure upon the injection of blood-derived MSCs (Manshadi *et al*., 2019). These results were in line with of our findings and those of other previous studies (Liu *et al*., 2014a; Sheikhansari *et al*., 2018). There is also some evidence regarding the positive effect of MSC on the repair of damaged tissues as MSCs could secrete cytokines and induce the secretion of bioactive molecules (Lai *et al*., 2014; 2015). They also play a critical role in the proliferation and regeneration of damaged tissues, as they contain fibroblast growth factor-2 which is a crucial factor in these regards (Wang *et al*., 2017). MSCs have shown great potential in regenerative medicine due to their advantages, such as abundant sources, easy access, multidirectional differentiation, and low immunogenicity and are safe. There are no side effects associated with stem cell therapy (Gao *et al*., 2022). In very rare cases: a slight increase in temperature after the procedure may happen; slight redness at the injection site has been reported. In this study only one patient had redness at the injection site, and after two hours it was resolved without any intervention.

The current study is one of the first attempts to evaluate the efficacy of blood-derived MSCs on women with POF as a pilot study. Our study was unique, because the study samples were human and we evaluated the critical hormones representing the overall ovarian function. However, our findings must be interpreted in light of our limitations, such as a small study group. In conclusion, our study indicated that blood-derived MSCs could significantly improve hormone secretion in women with POF. Implantation of MSCs in women with POF background was associated with an increase in AMH and AFC; while it reduced the serum level of E2 and FSH. MSCs could also cause menstrual restoration and pregnancy in women with POF. Further studies are required.

### Limitations

The main limitations include the small sample size.

## Data Availability

All supporting data are available through the corresponding author.
